# Supplementary figures and images for: Enhanced RNA replication and pathogenesis in recent SARS-CoV-2 variants harboring the L260F mutation in NSP6
Source: PLoS Pathog. 2025 Mar 31;21(3):e1013020. doi: 10.1371/journal.ppat.1013020 (PMC11981139; doi:10.1371/journal.ppat.1013020)

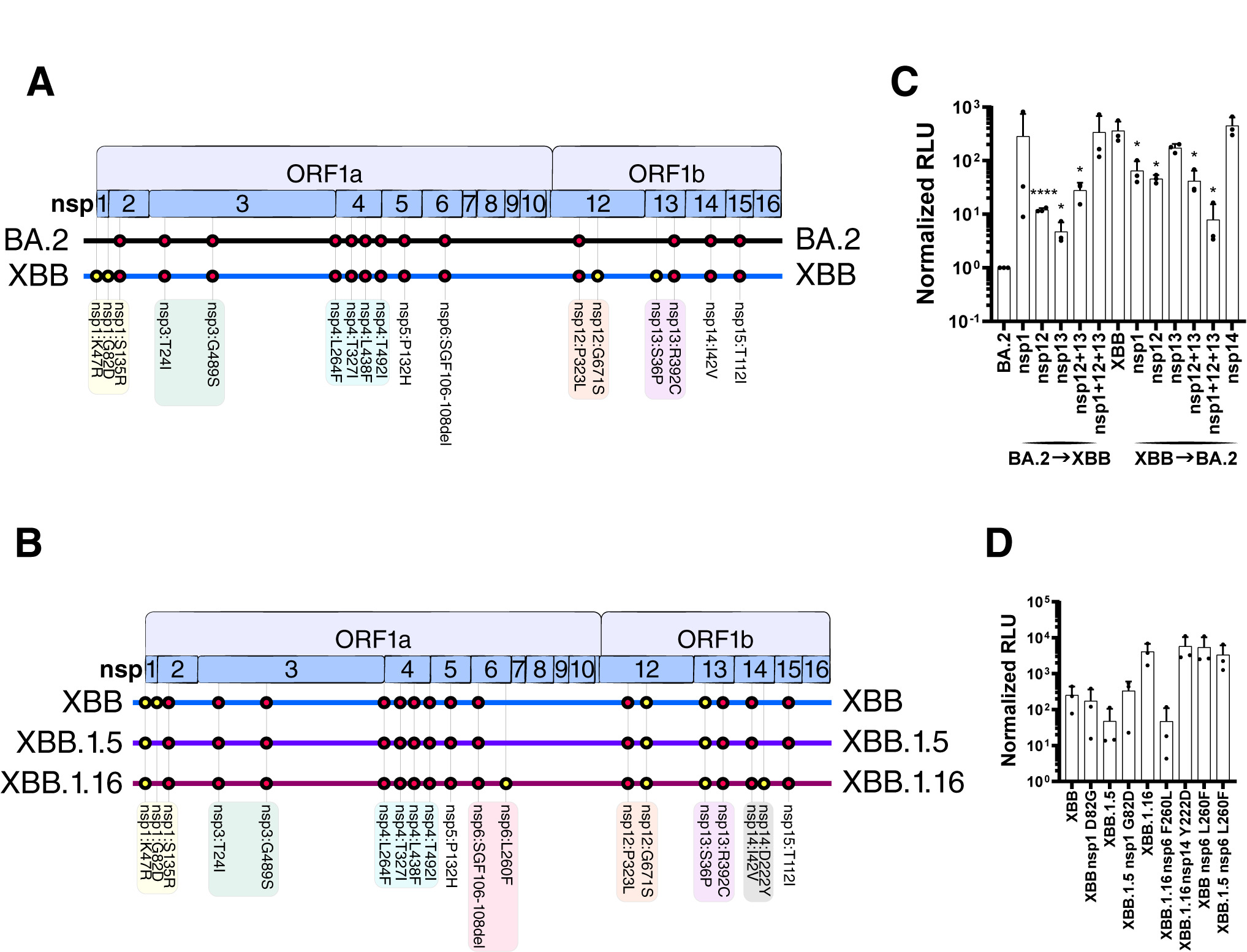

Supplement: S1 Fig — A and B) Consensus ORF1ab mutations (>95% of sequences at time of emergence) between BA.2 and XBB (A) and between the XBB-derived variants (B). BA.2 fixed mutations are indicated in red. C and D) Viral RNA replication was measured in infected VAT cells with indicated replicons and plotted as mean +/- SD of three independent biological replicates each conducted in triplicate. *, p<0.05; **, p<0.01; ***, p<0.001; ****, p<0.0001 by two-sided Student’s T-test. (TIF) [file ppat.1013020.s001.tif]

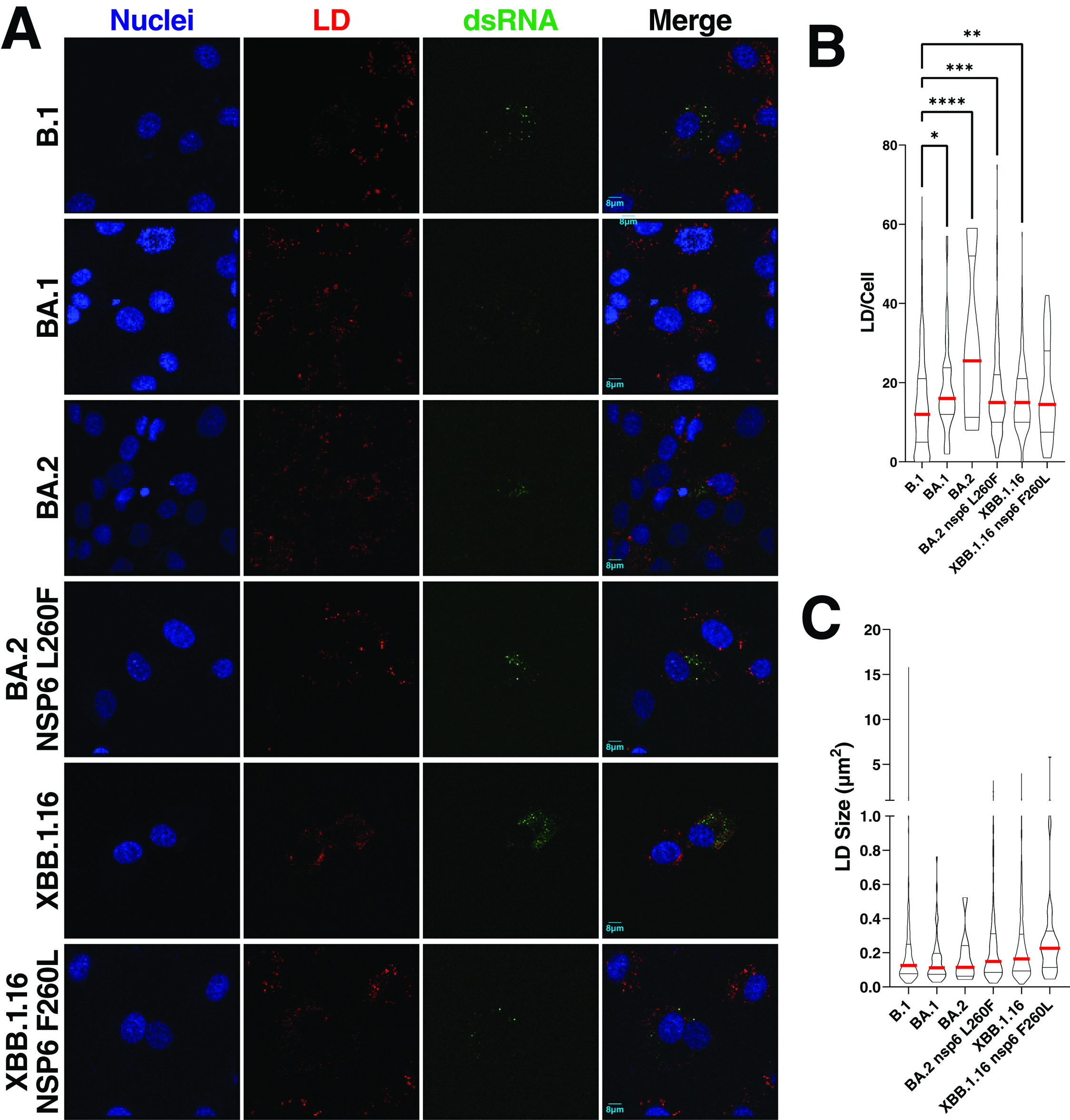

Supplement: S2 Fig — Representative immunofluorescence images of VAT cells infected with indicated replicons and stained at 24 hours post-infection for LD and dsRNA. Quantification of these images is presented in Fig 3C. (TIF) [file ppat.1013020.s002.tif]

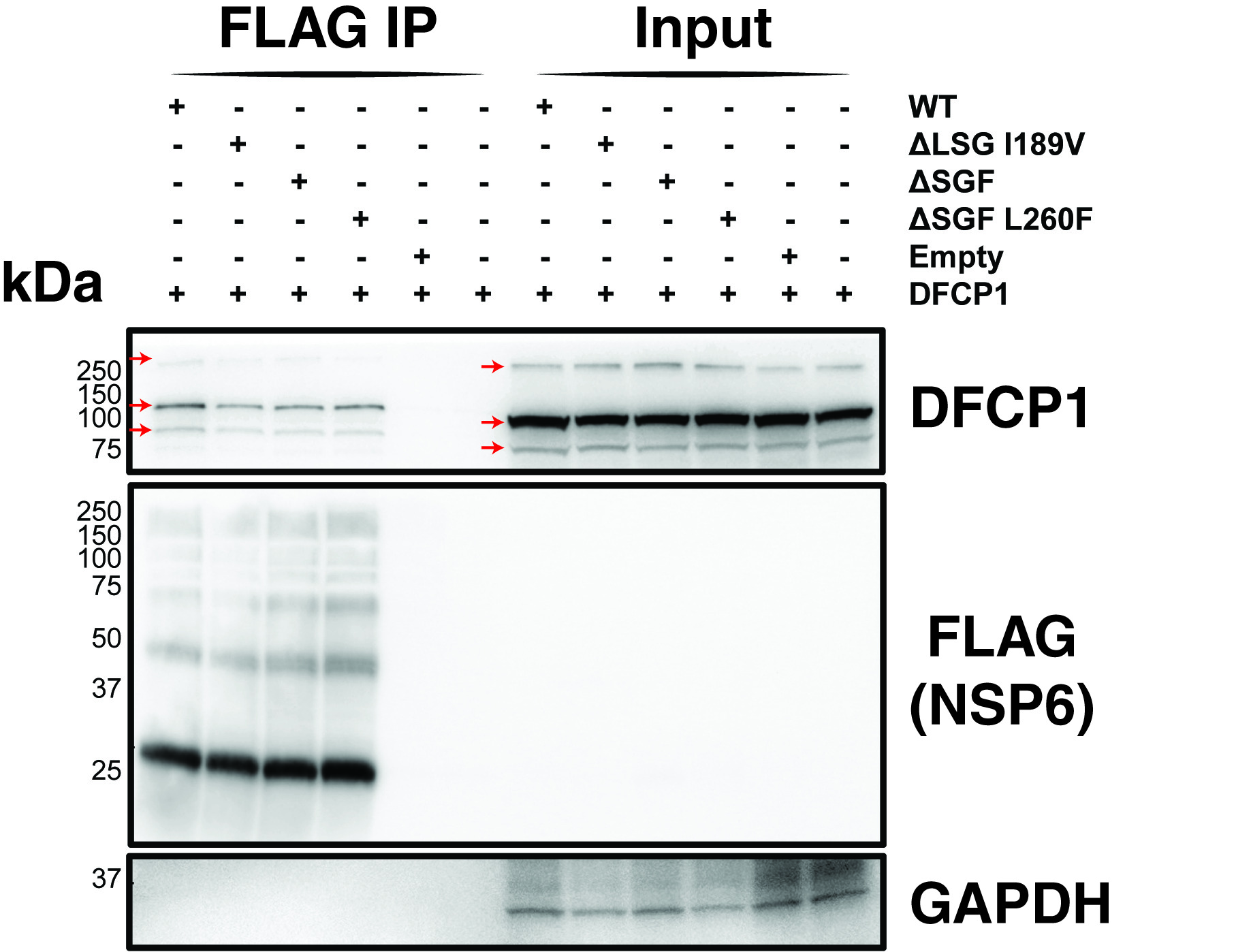

Supplement: S3 Fig — Western blot analysis of FLAG-NSP6 immunoprecipitation from cells co-transfected with DFCP1 and indicated NSP6 mutants. The left half of the blot indicates immunoprecipitated samples and the right half is the corresponding input samples. The blot is representative of two independent biological replicates. The red arrows indicate observed bands for DFCP1. (TIF) [file ppat.1013020.s003.tif]

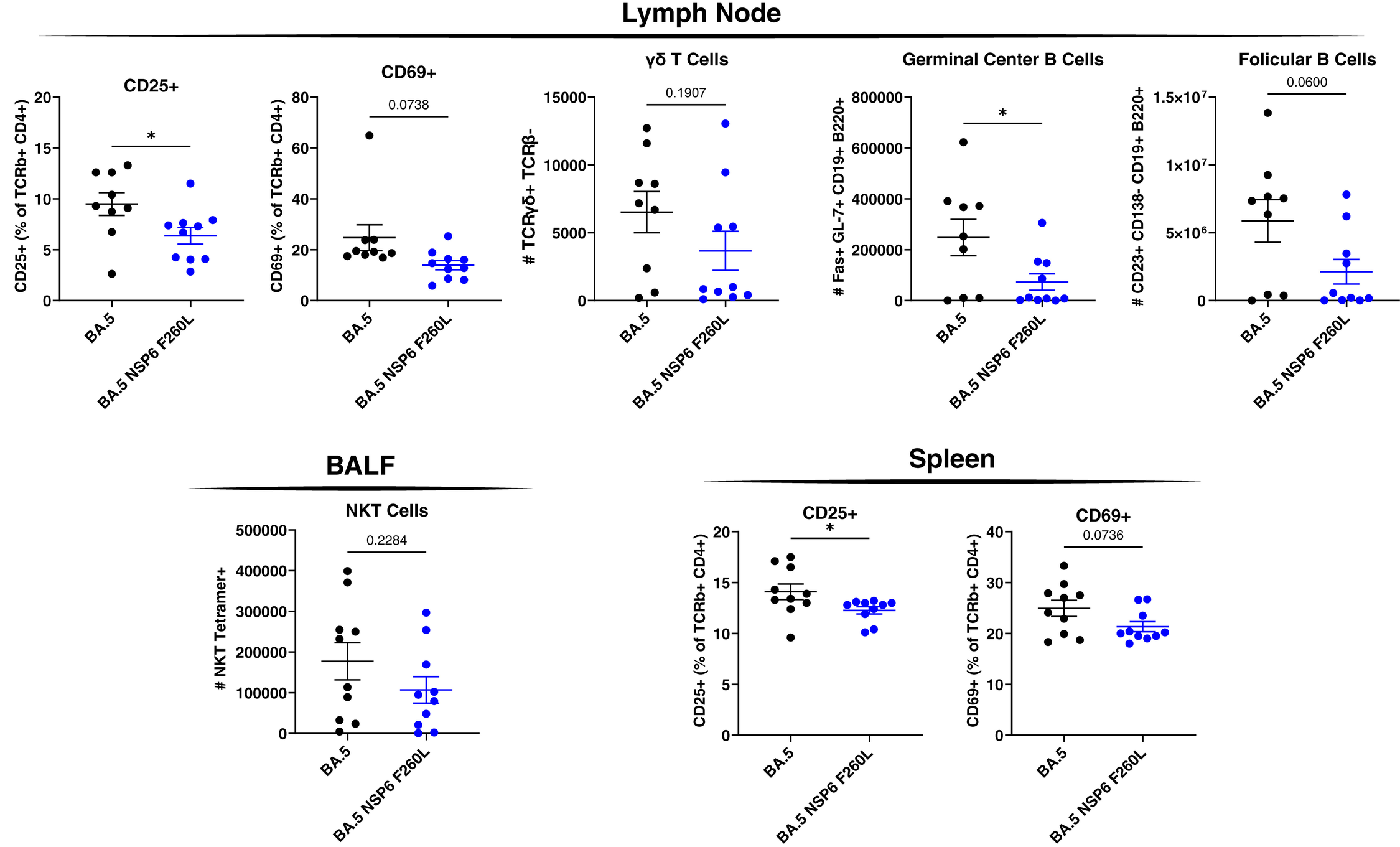

Supplement: S4 Fig — Flow cytometry analysis of indicated immune cell subsets in indicated organs was performed at the 5 days post-infection time-point of the in vivo experiment presented in Fig 4. The data is presented as mean +/- SD. *, p<0.05 by two-sided Student’s T-test. (TIF) [file ppat.1013020.s004.tif]

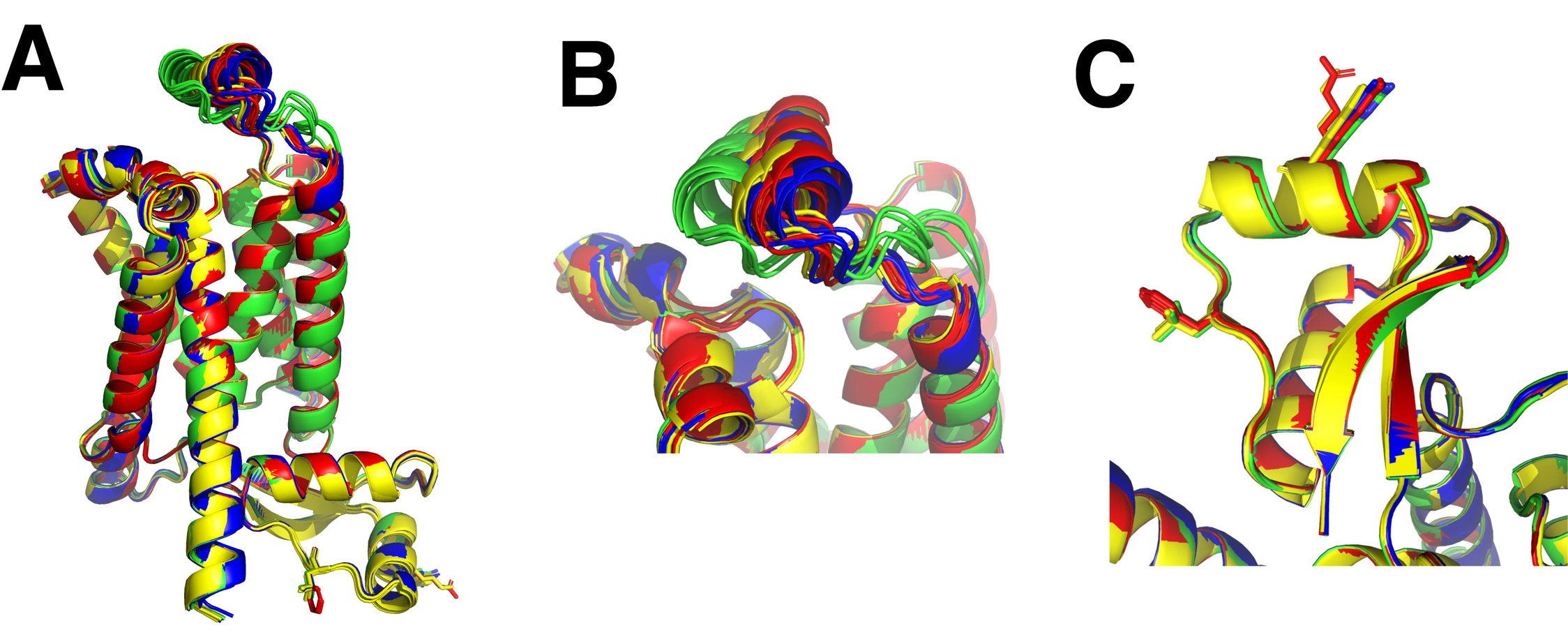

Supplement: S5 Fig — A) The top 4 predicted structures of NSP6 WT (green), ΔSGF (blue), ΔSGF/L260F (red), and V24F/ΔSGF/R252K (yellow) were aligned using PyMOL. B) Close up of the second alpha helix on the luminal side of the ER. C) Close up of the cytoplasmic tail of NSP6 with the amino acids at positions 252 and 260 drawn as sticks. (TIF) [file ppat.1013020.s005.tif]
