# Supplementary material for: Enhanced RNA replication and pathogenesis in recent SARS-CoV-2 variants harboring the L260F mutation in NSP6
Source: PLoS Pathog. 2025 Mar 31;21(3):e1013020. doi: 10.1371/journal.ppat.1013020 (PMC11981139; doi:10.1371/journal.ppat.1013020)
Supplement: Attachment — Submitted filename: NSP6 manuscript reviews_v4.docx [file ppat.1013020.s008.docx]

**Point-by-point response to Reviewers' Comments:**

**Part I - Summary**
Reviewer #1: This manuscript describes the role for a particular mutation in NSP6 L260F of SARS-CoV-2 on viral replication both in cultured cells and in a virus-susceptible mouse model. It was discovered that this mutation enhanced the replication of the viral RNA in both systems and this finding correlated with an enhanced host lipid droplet consumption by the mutated protein. In addition, this mutation also enhanced viral pathogenesis in mice compared to a F260L mutation in NSP6. This study provides novel and important insights by which non-spike proteins contribute to viral pathogenesis.

We thank the reviewer for pointing out that our paper provides “novel and important insights” and that we describe the important role of how non-Spike mutations impact replication and pathogenesis.

Reviewer #2: Taha and colleagues performed a comprehensive study on the evolution of SARS-CoV-2 variants, anlysinga mutations on the Spike and their impact on viral entry and replication. The authors also focused their analyses on NSP6, which is involved in viral RNA replication and lipid consumption. They report a negative correlation between viral entry and RNA replication: As variants evolved reduced entry functions due to immune pressure on Spike, RNA replication increased as a compensatory mechanism. They demonstrate that the frequent L260F mutation in NSP6 enhances viral infection in cells and increases pathogenesis in mice. The authors used a variety of experimental systems including novel tools, either Spike-defective or NSP6-defective genomes (replicons) of recent Omicron variants, which are clearly described and used. The study provides insights into evolutionary trajectories of recent variants and understanding of how the L260F mutation affects NSP6 function and viral replication. In summary, the manuscript demonstrates rigorous experimental techniques and provides novel insights into SARS-CoV-2 evolution.

We thank the reviewer for positively commenting on our “rigorous experimental techniques” and tools to comprehensively study SARS-CoV-2 evolution. We believe that they will broadly benefit the scientific community.

Reviewer #3: The study investigates the role of NSP6 mutations, particularly L260F, in SARS-CoV-2 RNA replication and on host cell lipid droplets (LD). Using a replicon-based assay, the authors show enhanced replication in variants carrying the L260F mutation and explore its potential effects on LD dynamics. However, while the approach is valuable, the proposed mechanism through which NSP6 L260F would exert its role in viral replication remains unsupported by the data, as specified below.

We thank the reviewer for calling our approach “valuable”. In the revised manuscript, we have made every effort to only present mechanisms that are supported by the presented evidence.

**Part II – Major Issues: Key Experiments Required for Acceptance**

Reviewer #1: (No Response)

Reviewer #2: I don’t have major concerns.
Minor point
1. The authors could discuss the more recent evolution of the JN.1 lineage. The predominant lineages are KP.3.1.1 and XEC. How the spike mutations will impact viral entry. Do these strains carry additional mutations in NSP6?

No, these strains do not carry additional mutations in NSP6. Based on reports (e.g. Liu et al. Lancet Infectious Diseases, Volume 25, Issue 1, e6 - e7), entry and immune evasion functions are enhanced in this lineage by additional mutations in the N-terminal domain (NTD) of the Spike protein. We have added this discussion in the revised manuscript (lines 139-141).

Reviewer #3:

Lipid droplets analysis

The authors report increased RNA replication in NSP6 L260F-containing variants such as BA.1, BA.2, and XBB.1.16 compared to wild-type NSP6. They show reduced mean fluorescence intensity (MFI) of LDs in these variants and conclude that enhanced LD consumption underlies the increased replication. However they do not mention other meaningful parameters, such as number and size of LDs. In addition, the reported differences in LD MFI are tiny and not fully consistent with the rate of RNA replication of the different virus variants. For instance, BA.1 exhibits the highest RNA replication, but its LD MFI value (400) is similar to that of BA.2 NSP6 L260F and XBB.1.16, which have one log lower replication rates. The lack of correlation between LD MFI and RNA replication undermines the proposed causative relationship. Furthermore, the differences in LD MFI are marginal (e.g., ~100 units) and accompanied by large data dispersion, raising questions about their biological relevance.

The authors use different clones stably expressing NSP6 mutants to analyze LDs, but the variability observed in LD measured parameters (also seen in the empty vector) makes it uncertain the interpretation of the reported tiny difference. Here the authors measure also the number and the size of LD: however no difference is measured in LD number and size between cells expressing wt NSP6 and cells transfected with the empty vector, reinforcing the suspect that the experimental system is not adequately set to assess LD dynamics. In addition the experimental design does not allow to discern whether the observed differences are due to altered LD biogenesis or LD consumption.

This is a recurrent theme brought up by this reviewer and we would like to address it in detail. First, we would like to address the reviewer’s concern that there is a lack of correlation across all variants between viral RNA replication and LD MFI in infected cells. We do not claim and have made every effort to remove any claims that viral RNA replication is exclusively dependent on NSP6. Viral RNA replication is influenced by many variables, NSP6 mutations being one of them. In fact, there are mutations in almost every viral protein across the different variants (See **Fig. 1A**), and mutations in NSP1, NSP12, and NSP13 directly impact RNA replication of XBB variants independently of NSP6 (See **Supp. Fig. S1A and S1C**). We show that mutations in NSP6 are driving the differences in LD MFI and only indirectly affect viral RNA replication, in the context of other mutations in viral proteins if present. Indeed, comparing variants of the same genotype different only in NSP6 mutations shows excellent correlation between viral RNA replication and LD MFI (See **Fig. 3B and 3C** comparing BA.2 vs. BA.2 nsp6 L260F and XBB.1.16 vs. XBB.1.16 nsp6 F260L). This has been further clarified in the revised text (line 151).

**Fig. 1A**

**Supp. Fig. S1A and S1C**

**Fig. 3B and 3C**

Second, we have now included LD numbers and size missing for the experiment in Fig. 3C in new **Supp. Fig. 2B and 2C**, for which we do not find significant differences in LD size**.** LD numbers per cell were higher in cells infected with all variants compared with B.1. This underscores our data in Fig. 3E-G where we also see the most pronounced changes in LD MFI but not in numbers or sizes.

**Supp. Fig. 2B and 2C**

Third, we would like to address the reviewer’s concern about the “marginal” differences and the “large data dispersion” of our LD data. Our observation that small, but significant, differences in LD MFI have marked consequences in biology are not unique to us but similar results have been widely published in the literature (Sci Transl Med. 2024 Feb 28;16(736):eadf9874. doi: 10.1126/scitranslmed.adf9874.; Prostate. 2024 May;84(7):644-655. doi: 10.1002/pros.24680.; Cancer Metab. 2020 Jun 19:8:11. doi: 10.1186/s40170-020-00217-6.; Commun Biol. 2023 Mar 21;6(1):300. doi: 10.1038/s42003-023-04682-9.). Part of the relatively large dispersion may be explained by the fact that we employ a high-throughput screening (HTS) microscope (Molecular Devices ImageXpress Micro Confocal High-Content Imaging system) to perform high-content imaging of cells infected with variant replicons. We used the granularity package of MetaXpress, which unbiasedly counts all LDs across an entire well but focuses only on infected or transduced cells. The experiments in Fig. 3B and 3C were conducted in Vero cells stably expressing ACE2 and TMPRSS2 (VAT); these cells have less and smaller lipid droplets compared with other cell types such as Huh-7.5 cells (compare LD MFI between **Fig. 3C and Fig. 3E**). Therefore, ~100 units difference in LD MFI in VAT cells is not only statistically, but also biologically significant as it represents a 25% change in LD content in the cells. Similar changes also apply to LD area as a difference in LD area of 25–35% between cells expressing NSP3/4 and cells expressing NSP3/4/6 was reported in Nature; these data also displayed large data dispersion (See **Fig. 4F** from Nature. 2022 Jun;606(7915):761-768. doi: 10.1038/s41586-022-04835-6.).

**Fig. 3C and 3E**

**Fig. 4F from** Nature. 2022 Jun;606(7915):761-768.

Fourth, we would like to address the reviewer’s concern about “LD number and size between cells expressing wt NSP6 and cells transfected with the empty vector.” We measured LD MFI, numbers, and size per transduced cell and plotted them using box and whisker plot to better visualize mean, range, and interquartile range between the cells expressing NSP6 and the empty vector control (See Fig. **3E-G**). Statistical comparison of all LD data indicate that the strongest effect is again on LD MFI and not size and number. We agree that the experimental setup is not designed to measure altered LD biogenesis vs. LD consumption and have changed the language in the manuscript and added a discussion starting on line 287. However, our experimental system allows us to say that cells expressing NSP6 harboring L260F have significantly less LD lipid content compared with cells expressing the empty vector control (See **Fig 3E**).

**Fig. 3E-G**

Additionally, NSP6 expression is not adequately controlled across the clones. In Fig. S4, large fields of NSP6-expressing clones show a low percentage of NSP6-positive cells, raising questions about whether mixed populations were used and the expression levels of NSP6 in these clones.

It is important to note that the cells in **Fig. 3D** are lentivirally transduced cells stably expressing NSP6 and all have uniformly high expression of NSP6. We have removed Supp. Fig. S4, which had 293T cells transfected with NSP6 and Cyb5-EGFP and have mixed expression if any. Regardless of the cells used, we have only measured the LD properties in cells expressing high amounts of NSP6 as we perform FLAG staining during microscopy. We do not measure LD content in cells that are not expressing NSP6 and do not incorporate such data into our analyses.

**Fig. 3D**

The authors fail to provide individual LD measurement values, but the graphs reveal substantial data dispersion (Fig. 3E-3F), while the differences among mutants are minimal (e.g., Fig. 3F). These small differences, even if statistically significant, seem unlikely to represent biologically meaningful changes. Further, in Fig. 3G, neither the graph’s scale nor the unit of measurement is provided, hindering interpretation of the data. These omissions and inconsistencies undermine the robustness of the authors’ conclusions regarding NSP6’s impact on LDs.

We respectfully disagree. Small differences can represent biologically meaningful changes. We have already pointed to small differences in LD biology featured in the recent Nature paper (See **Fig. 4F** from Nature. 2022 Jun;606(7915):761-768. doi: 10.1038/s41586-022-04835-6.) and similar variability in LD sizes and MFI have been observed in numerous publications (Sci Transl Med. 2024 Feb 28;16(736):eadf9874. doi: 10.1126/scitranslmed.adf9874.; Prostate. 2024 May;84(7):644-655. doi: 10.1002/pros.24680.; Cancer Metab. 2020 Jun 19:8:11. doi: 10.1186/s40170-020-00217-6.; Commun Biol. 2023 Mar 21;6(1):300. doi: 10.1038/s42003-023-04682-9.). Last year, Victor Ambros and Gary Ruvkun received the Nobel Prize in Physiology or Medicine for the discovery of microRNAs, highlighting how small molecular changes can have profound effects on gene regulation and biological functions.

We thank the reviewer for pointing out the missing unit (μm^2^) in **Fig. 3G** which we have provided in the revised figure. The graph scale has been corrected to indicate that it’s log scale. Our average LD size range from 0.5-5 μm^2^, which again is within published values of LDs (see list of publications above).

**Fig. 4F from** Nature. 2022 Jun;606(7915):761-768.

**Fig. 3E-G**

As in the case of cells infected with virus variants, also in the mutant NSP6 expressing cells only a correlative evidence is, at best, presented and no causative link is either explored or demonstrated.

We constructed mutant recombinant viruses with a single point mutation in NSP6 on the same exact genetic background to validate that this single point mutation alters viral replication (**Fig. 3B**) and LD content (**Fig. 3C**). These rigorous validation experiments mechanistically link the mutation in NSP6 to infection and lipid droplet biology and excludes that other mutations or alterations in viral or host proteins outside of NSP6 affect lipid droplet content and levels of viral replication in infected cells. We would like to point out that while this reviewer consistently doubts statistically significant data, reviewer #1 calls the same data “striking”.

**Fig. 3B and 3C**

**Fig. 3E**

ER Zippering and DMVs morphology
The authors conclude that L260F neither affects the ER zippering activity of NSP6 nor impacts the DMV biogenesis, but the data are insufficient to support this conclusion. The EM images in Figure 4b are low-resolution and fail to clearly depict DMVs or zippered ER membranes. Quantitative analysis of these structures is absent, making it impossible to draw meaningful comparisons. Higher resolution EM images and a more comprehensive analysis of DMV morphology are needed.

We agree that the EM images are low-res. As they do not add significant additional information to the current manuscript, we have removed the EM images and the ER zippering analysis.

DFCP1-NSP6 interaction
In the co-IP experiments examining DFCP1-NSP6 interactions, the western blots show different molecular weights for DFCP1 in the input compared to the IP samples, as well as multiple bands for NSP6 that do not correspond to its expected ~35 kDa size. These anomalies are not explained, raising concerns about the identity of the observed bands. Hence, the authors claim no differences in DFCP1 interactions between NSP6 mutants and wild-type, but the data presented are insufficient to draw this conclusion.

We apologize that the previous figures were not satisfying for the reviewer. We have repeated the experiment and updated the WB image in **Supp. Fig. S3**. We have also now clearly marked the three detected bands for DFCP1 (see red arrows) in both input and IP samples and now discuss how their relative migration differences are proportional indicating that the entire lane migrated differently and not just DFCP1 migrating at a different rate.

In addition, we provide unaltered and uncropped WB images to show the entire migration pattern for NSP6 with empty vector controls validating the identity of the band as NSP6. Notably, our WB data for NSP6 look very similar to that shown for NSP6 antibodies (<https://www.thermofisher.com/antibody/product/SARS-CoV-2-NSP6-Antibody-Polyclonal/PA5-120714> and <https://www.novusbio.com/products/sars-cov-2-nsp6-antibody_nbp3-16000?srsltid=AfmBOooKHdKOgBitzzd-UsX2jaPNV7RFEKFrCUlD2sMC9B4Aj7nZUzCp>) and in the literature (mBio. 2022 Aug 30;13(4):e0097122. doi: 10.1128/mbio.00971-22. and EMBO J. 2021 Sep 1;40(17):e107776. doi: 10.15252/embj.2021107776).

**Supp. Fig. S3**

**Fig. 1B from mBio. 2022 Aug 30;13(4):e0097122.**

**Fig. EV1A from EMBO J. 2021 Sep 1;40(17):e107776.**

**Part III – Minor Issues: Editorial and Data Presentation Modifications**

Reviewer #1: Comments:

1. Both NSP1 and NSP6 affect viral RNA abundances. Explain for the more general reader the mechanistic differences by which these two proteins modulate viral gene expression.

We thank the reviewer for suggesting this, and we have added a description of the function of NSP1 starting on line 156.

2 Fig. 2B,C. XBB seems to display enhanced RNA replication., Can you comment on its genotype in relation to XBB.1.6 and BQ.1.1?

We thank the reviewer for pointing this out. We have conducted comprehensive analysis of mutations between these different genotypes and how they relate to XBB and provided the results in Fig. S1 and commented on it in line 151.

3. Fig. 3D. Panels 2-4 are difficult to read. Provide a higher resolution figure.

We have provided a higher resolution Fig. 3D to address this.

4. The phenotype of NSP6 L60F versus NSP6 F260L is striking. Can the authors provide a molecular docking model which may give some clues?

We thank the reviewer for this suggestion. We predicted the top 4 structures of all nsp6 mutants using AlphaFold and found that the most relevant structure altering mutation is deltaSGF which shifts the position of the second alpha helix on the luminal side of the ER (See **Supp. Fig. 6**: WT - Green, BA.2 - Blue, XBB.1.16 - Red, and JN.1.1.1 - Yellow). However, the mutations R252K and L260F lie in the terminal cytosolic portion of NSP6 and do not appear to alter structure, but likely alter interactions with other proteins. We have provided the structural analysis in new **Supp. Fig. 6** and added discussion in the text on line 265.

**Supp. Fig. 6**

Reviewer #2: (No Response)

Reviewer #3: (No Response)
